# Human amnion-derived mesenchymal stem cells attenuate xenogeneic graft-versus-host disease by preventing T cell activation and proliferation

**DOI:** 10.1038/s41598-021-81916-y

**Published:** 2021-01-28

**Authors:** Yoshiyuki Tago, Chiho Kobayashi, Mineko Ogura, Jutaro Wada, Sho Yamaguchi, Takashi Yamaguchi, Masahiro Hayashi, Tomoyuki Nakaishi, Hiroshi Kubo, Yasuyoshi Ueda

**Affiliations:** 1 Biotechnology Research Laboratories, Kaneka Corporation, 1-8, Miyamae-cho, Takasago-cho, Takasago, Hyogo 676-8688 Japan; 2 Regenerative Medicine and Cell Therapy Laboratories, Kaneka Corporation, Kobe, Japan

**Keywords:** Experimental models of disease, Mesenchymal stem cells, Graft-versus-host disease

## Abstract

Acute graft-versus-host disease (GVHD) is characterized by severe tissue damage that is a life-threatening complication of allogeneic hematopoietic stem cell transplantation. Due to their immunosuppressive properties, mesenchymal stem cells (MSC) have been increasingly examined for the treatment of immune-related diseases. We aimed to assess the immunosuppressive effects of human amnion-derived MSC (AMSC) in a xenogeneic GVHD NOD/Shi-scid IL2rγnull mouse model using human peripheral blood mononuclear cells (PBMC). Additionally, we used human bone marrow-derived MSC (BMSC) as comparative controls to determine differences in immunomodulatory functions depending on the MSC origin. Administration of AMSC significantly prolonged survival, and reduced human tumor necrosis factor-α (TNF-α) concentration and percentage of programmed cell death protein-1 receptor (PD-1)^+^CD8^+^ T cell populations compared with in GVHD control mice. Furthermore, colonic inflammation score and percentage of human CD8^+^ T cell populations in AMSC-treated mice were significantly lower than in GVHD control and BMSC-treated mice. Interestingly, gene expression and protein secretion of the PD-1 ligands were higher in AMSC than in BMSC. These findings are the first to demonstrate that AMSC exhibit marked immunosuppression and delay acute GVHD progression by preventing T cell activation and proliferation via the PD-1 pathway.

## Introduction

Acute graft-versus-host disease (GVHD) is characterized by severe tissue damage that is a life-threatening complication of allogeneic (allo) hematopoietic stem cell transplantation (HSCT). Donor lymphocytes attacking various host tissues can lead to cutaneous inflammation, intractable diarrhea, hepatobiliary disease, pulmonary fibrosis, and musculoskeletal injury^1^.

In Japanese patients with grade II‒IV acute GVHD, response rates to corticosteroids, which are the first-line treatment for acute GVHD, range from 40 to 70%^2^. However, patients without response to corticosteroid therapy have a dismal long-term prognosis, with an overall survival rate of only 5‒30%^3^. Anti-thymocyte globulin as second-line treatment for steroid-refractory acute GVHD have been reported to be associated with an increased incidence of infectious disease in a meta-analysis^4^. Recently, ruxolitinib, a selective Janus kinase (JAK1 and JAK2) inhibitor showed potential efficacy in phase 2 and 3 trials, and the Food and Drug Administration approved for use in patients 12 years of age or older who had steroid-refractory acute GVHD^5^. Considering drug resistance and relapse, more treatment options are desirable.

Mesenchymal stem or stromal cells (MSC) isolated from multiple tissues such as bone marrow, adipose, and perinatal tissues are recognized for their potential immunomodulatory effects^6^. Importantly, bone marrow-derived MSC (BMSC) have been introduced as a cell-based therapy for acute GVHD^7^ and registered as regenerative medicine for steroid-refractory acute GVHD in Japan^8^. Although the cytokine-mediated effects of MSC occur primarily via immunomodulation, the underlying cellular and molecular mechanisms remain unclear.

The advantages of utilizing amnion, a perinatal tissue normally discarded after delivery, as a stem cell source include non-invasive procurement and vast abundance of source material. Additionally, the immunomodulatory characteristics of mesenchymal cells within fetal membranes play key roles in fetal-maternal tolerance^9^. Several recent studies reported that human amnion-derived MSC (AMSC) exhibited therapeutic efficacy in rodent disease models including colitis induced by chemicals^10,11^, heart failure induced by coronary artery ligation^12^, and allo-GVHD^13^.

Immunodeficient mice engrafted with human cells and tissues, commonly known as humanized mice, are used for medical research on various human diseases^14–16^. NOD/Shi-scid IL2rγ^null^ (NOG) mice lack T, B, and NK cells, and have macrophages and dendritic cells with reduced functions. These mice exhibit a higher level of immunodeficiency. Ito et al. ^17^ reported a novel xenogeneic (xeno) GVHD model using NOG mice and human peripheral blood mononuclear cells (PBMC) in the absence of total body irradiation. Xeno-GVHD mice are suitable animal models to elucidate the immunoregulatory properties of human MSC in vivo prior to their assessment in clinical trials.

Several studies have indicated that the immunosuppressive function of MSC in xeno-GVHD mice might depend on the MSC origin^18–20^. Identifying more effective MSC with improved immunomodulation from different sources can considerably contribute to cell therapy.

We elucidated the immunosuppressive effects of AMSC in a xeno-GVHD mouse model in vivo and in a mitogenic stimulation model in vitro. Additionally, we used BMSC as comparative controls to determine differences in immunomodulatory functions depending on the MSC origin.

## Results

### AMSC have an enhanced potency to treat xeno-GVHD mice: long-term experiment

**Figure 1:**
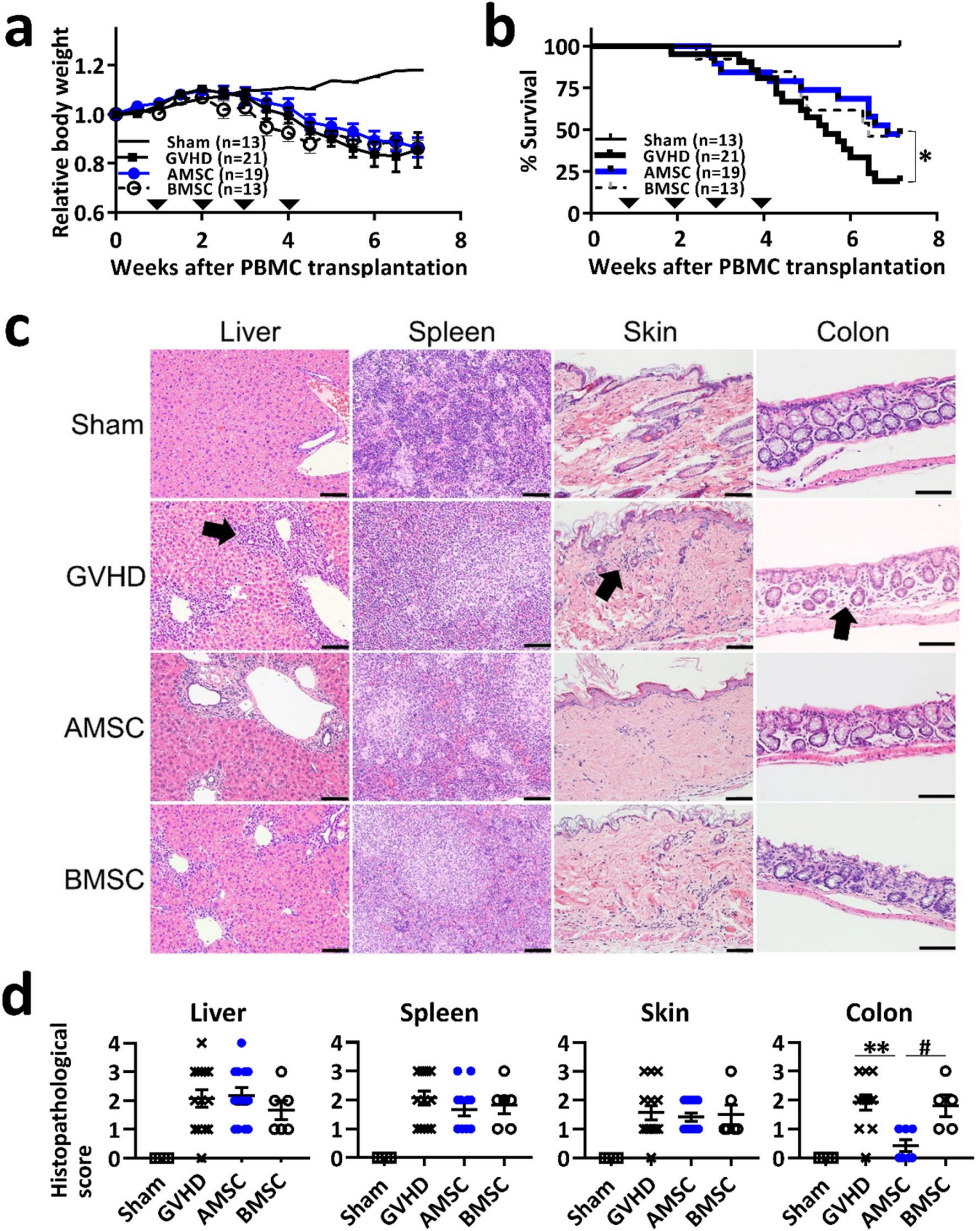
AMSC have immunosuppressive effects in xeno-GVHD mice. GVHD was induced by transplantation of 3 × 10^6^ cells/mouse PBMC administered intravenously on week 0. Mice were subsequently administered 8 × 10^6^ cells/kg AMSC (n = 19) or BMSC (n = 13) intravenously once a week from week 1 to 4 (inverted triangle). Sham (n = 13) and GVHD control (n = 21) mice were administered vehicle. In this long-term experiment, survived mice in all groups were sacrificed at week 7. Combined data from two or three independent experiments are represented. (**a**,**b**) Relative body weight and survival rate. (**c**) H&E staining of liver, spleen, skin, and colon tissues. Mononuclear cell infiltration (↑) are represented in GVHD target organs. Magnification, × 200. Scale bar, 50 μm. (**d**) Histopathological scores of liver, spleen, skin, and colon tissues. Data are represented as mean ± SEM. Each symbol represents an individual mouse. ^#^*P* < 0.05 AMSC versus BMSC; *P < 0.05, **P < 0.01 AMSC versus GVHD.

Human AMSC have been reported to improve changes in body weight in a mouse model of allo-GVHD^13^. Thus, we first determined whether human AMSC administration had an immunosuppressive effect on mice with severe xeno-GVHD induced by human PBMC^17,21^ compared with human BMSC administration^22^. All GVHD induction groups started to decrease body weight at around week 2 (Fig. 1a). At week 7, about 80% mice of GVHD controls died (Fig. 1b). AMSC treatment, but not BMSC treatment, significantly improved mortality compared with GVHD controls (*P* < 0.05), although the difference was not significant between AMSC and BMSC treatment in this regard (Fig. 1b). Histopathologically, mononuclear cell infiltration was observed in the target organs of xeno-GVHD mice (Fig. 1c). Severe inflammation was observed particularly in the liver and spleen tissues in all GVHD induction groups. Colonic inflammation score was significantly lower in AMSC-treated mice than in GVHD controls and BMSC-treated mice (*P* < 0.01 and *P* < 0.05, respectively) (Fig. 1d). These data suggested that AMSC exhibited marked immunosuppression and delayed GVHD progression.

### AMSC inhibit the activation and proliferation of human CD8^+^ T cells in xeno-GVHD mice: middle-term experiment

**Figure 2:**
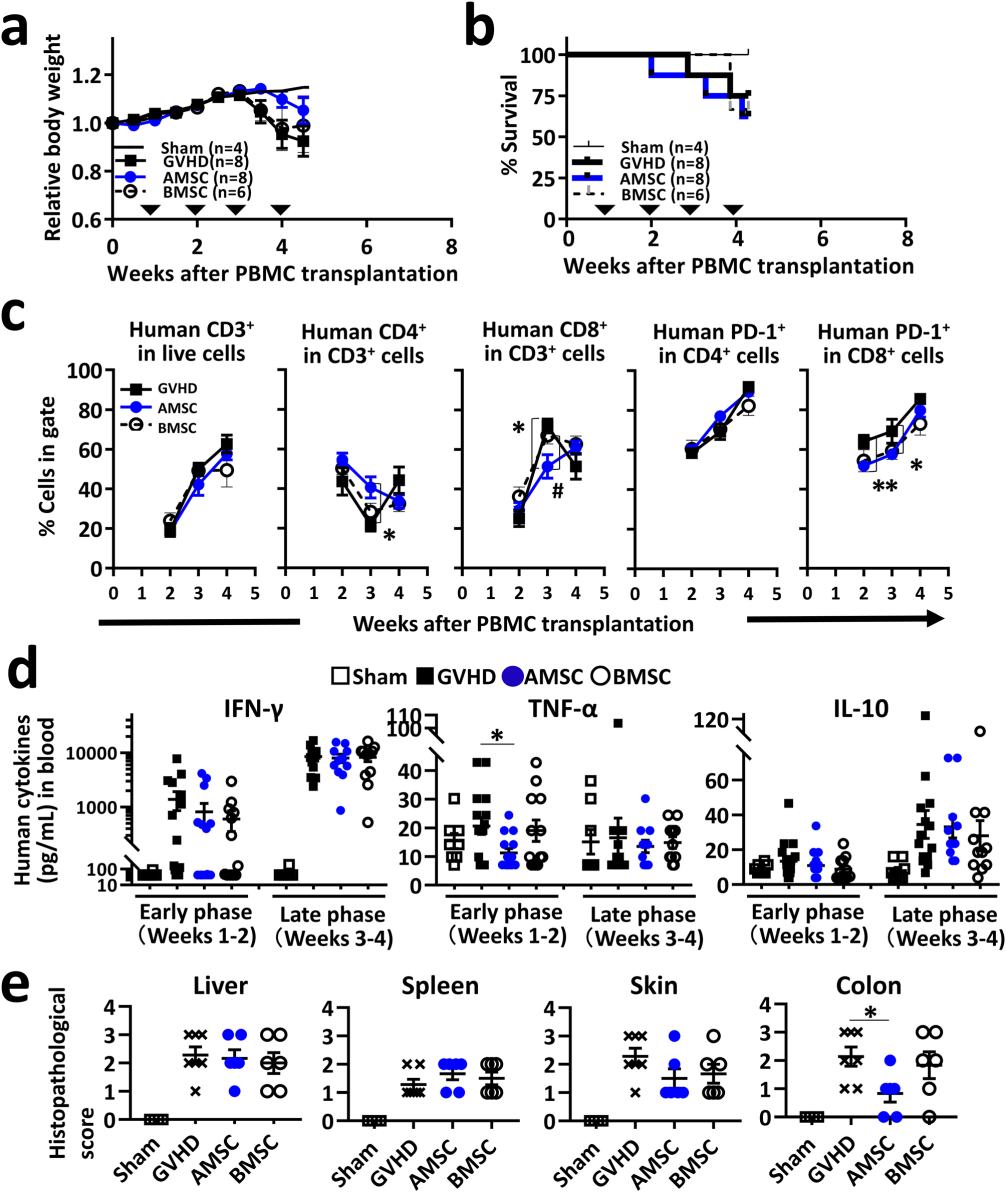
AMSC reduce the activity of human CD8^+^ T cells in xeno-GVHD mice. GVHD was induced by transplantation of 3 × 10^6^ cells PBMC/mouse administered intravenously on week 0. Mice were administered 8 × 10^6^ cells/kg AMSC (n = 8) or BMSC (n = 6) intravenously once a week from week 1 to 4 (inverted triangle). Sham (n = 4) and GVHD control (n = 8) mice were administered vehicle. In this middle-term experiment, survived mice were sacrificed at week 4. Representative data from one experiment shown. (**a**,**b**) Relative body weight and survival rate. (**c**) Human lymphocyte subpopulation in the peripheral blood of mice analyzed by flow cytometer at indicated time points. Populations of CD4^+^ or CD8^+^ in gated CD3^+^ T cells, and population of PD-1^+^ in gated CD4^+^ or CD8^+^ T cells were calculated. (**d**) Multibead-based immunoassay data of human IFN-γ, TNF-α, and IL-10 concentrations in the plasma collected in the early (weeks 1‒2) and late (weeks 3‒4) phases. (**e**) Histopathological scores of liver, spleen, skin, and colon tissues. Data are represented as mean ± SEM. Each symbol represents an individual mouse. *P < 0.05, **P < 0.01 AMSC versus GVHD; ^#^*P* < 0.05 AMSC versus BMSC.

To evaluate the mechanism underlying acute GVHD suppression by human AMSC, we analyzed the temporal changes in human lymphocyte subpopulation in the blood of xeno-GVHD mice. In this middle-term experiment, there were no significant differences in relative body weight or survival in any of GVHD induction groups (Fig. 2a,b). Percentage of human CD3^+^ T cell populations increased from weeks 2 to 4, finally reaching 50‒60% in all GVHD induction groups (Fig. 2c). Percentage of human CD4^+^ T cell populations were significantly higher in AMSC-treated mice than in GVHD controls (*P* < 0.05), although the difference was not significant between AMSC and BMSC treatment in this regard. Conversely, percentage of human CD8^+^ T cell populations was significantly lower in AMSC-treated mice than in GVHD controls and BMSC-treated mice (*P* < 0.05), respectively, at week 3.

We also measured programmed cell death protein-1 receptor (PD-1), which is an activation marker of T cells and expressed in multiple GVHD target organs^23^. Percentage of human PD-1^+^CD8^+^ T cell populations was significantly lower in AMSC-treated mice than in GVHD controls at weeks 2 and 3 (*P* < 0.01 and *P* < 0.05, respectively), although the difference was not significant between AMSC and BMSC treatment in this regard.

To elucidate the contribution of soluble factors to immunosuppression, we measured human cytokine concentrations in the plasma of xeno-GVHD mice (Fig. 2d). Interferon-γ (IFN-γ) exhibited a time-dependent increase consistent with acute GVHD progression. However, there were no alterations associated with AMSC or BMSC administration compared with GVHD controls. Tumor necrosis factor-α (TNF-α) concentrations were higher in the early phase than in the late phase in this experiment, in contrast to the detected changes in IFN-γ concentrations. Interestingly, AMSC treatment, but not BMSC treatment, prevented the increase in TNF-α concentrations by a significant difference compared with GVHD control (*P* < 0.05). No differences were observed in IL-10 concentrations among GVHD induction groups. Histopathologically, AMSC treatment, but not BMSC treatment, reduced the colonic inflammation score by a significant difference compared with GVHD controls (*P* < 0.05) (Fig. 2e), which almost matches the results of the long-term experiment (Fig. 1e). These results suggested the ability of AMSC to inhibit proliferation of CD8^+^ T cells was significantly higher than that of BMSC, although AMSC and BMSC had the similar ability to inactivate of CD8^+^ T cells in xeno-GVHD mice.

### AMSC modulate the cytokines and chemokines production by preventing T cell activation and proliferation in PHA/IL-2-stimulated PBMC in vitro

**Figure 3:**
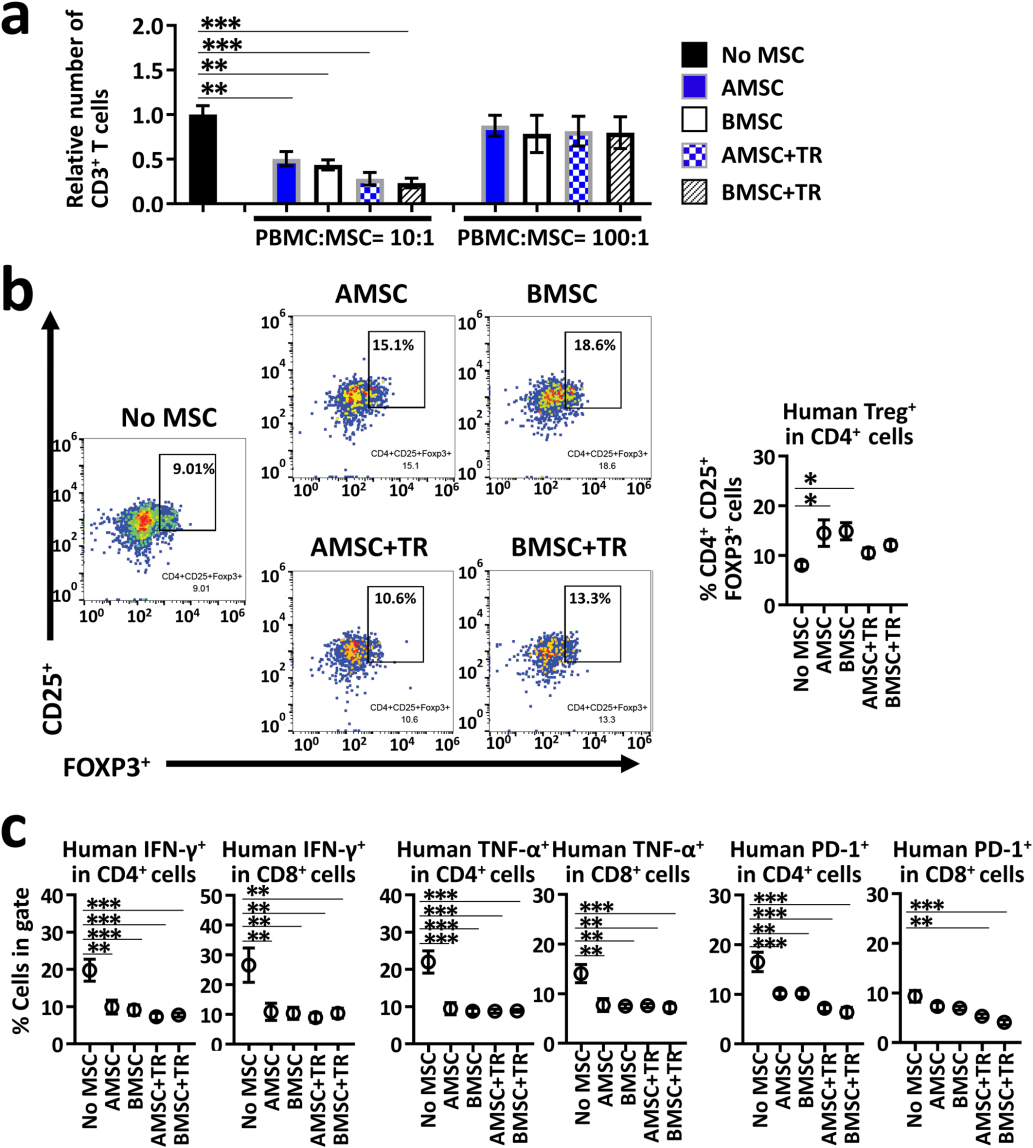
AMSC exert immunomodulatory effects in PHA/IL-2 stimulated PBMC in vitro. PBMC were stimulated with 4 μg/mL PHA and 100 U/mL IL-2 for 72 h with or without AMSC or BMSC at a PBMC/MSC ratio of 10:1 (**a**‒**c**) or 100:1 (**a**). For indirect coculture experiments, a trans well (TR) system comprising upper wells with PBMC and lower wells with MSC was used. Combined data from more than three independent experiments are presented as mean ± SEM. (**a**) Relative number of CD3^+^ T cells shown as 1 for No MSC. Unstimulated PBMC control showed 0.21 in this assay. (**b**,**c**) Population of human Treg (CD25^+^FOXP3^+^) in CD4^+^ T cells, and population of human T cells expressing IFN-γ, TNF-α, and PD-1 in human CD4^+^ or CD8^+^ T cells analyzed by flow cytometer. Unstimulated PBMC control showed 5.6% Treg, 2.3% IFN-γ^+^CD4^+^, 3.3% IFN-γ^+^CD8^+^, 3.2% TNF-α^+^CD4^+^, 3.6% TNF-α^+^CD8^+^, 5.2% PD-1^+^CD4^+^, 5.5% PD-1^+^CD8^+^ in each measurement. * *P* < 0.05, ** *P* < 0.01, *** *P* < 0.001 versus No MSC.

We used an in vitro model to recapitulate the microenvironment in xeno-GVHD mice, and examined the mechanisms underlying human AMSC-mediated immunosuppression. We utilized phytohemagglutinin (PHA) as a mitogen, IL-2 as a proliferation inducer^24^, and T cells in human PBMC as responders. To examine whether the immunomodulatory effect of AMSC need cell–cell contact or not, we used a transwell system for indirect coculture to separate T cells and MSC. By stimulating with PHA/IL-2 related to T cell activation and proliferation, numbers of relative CD3^+^ T cell, and percentages of regulatory T cells (Treg; CD4^+^CD25^+^forkhead box P3 (FOXP3)^+^), and T cells (CD4^+^ or CD8^+^) expressing IFN-γ, TNF-α, and PD-1 populations were increased in PHA/IL-2-stimulated PBMC (No MSC) compared with in unstimulated PBMC controls (data shown in Fig. 3 legend).

PBMC cocultured with AMSC or BMSC exhibited a concentration-dependent, significant reduction in CD3^+^ T cell proliferation compared with No MSC (*P* < 0.01 and *P* < 0.05) (Fig. 3a). Furthermore, Treg population was significantly higher in PBMC directly cocultured with MSC than in No MSC (*P* < 0.05), although it was not in PBMC indirectly cocultured with MSC (Fig. 3b). Conversely, populations of T cells expressing IFN-γ, TNF-α, and PD-1 were significantly lower in both direct and indirect PBMC–MSC cocultures than in No MSC (*P* < 0.001 and *P* < 0.01) (Fig. 3c). These data indicated that there were no differences in the ability to inactivate effector T cells between AMSC and BMSC in vitro.

**Figure 4:**
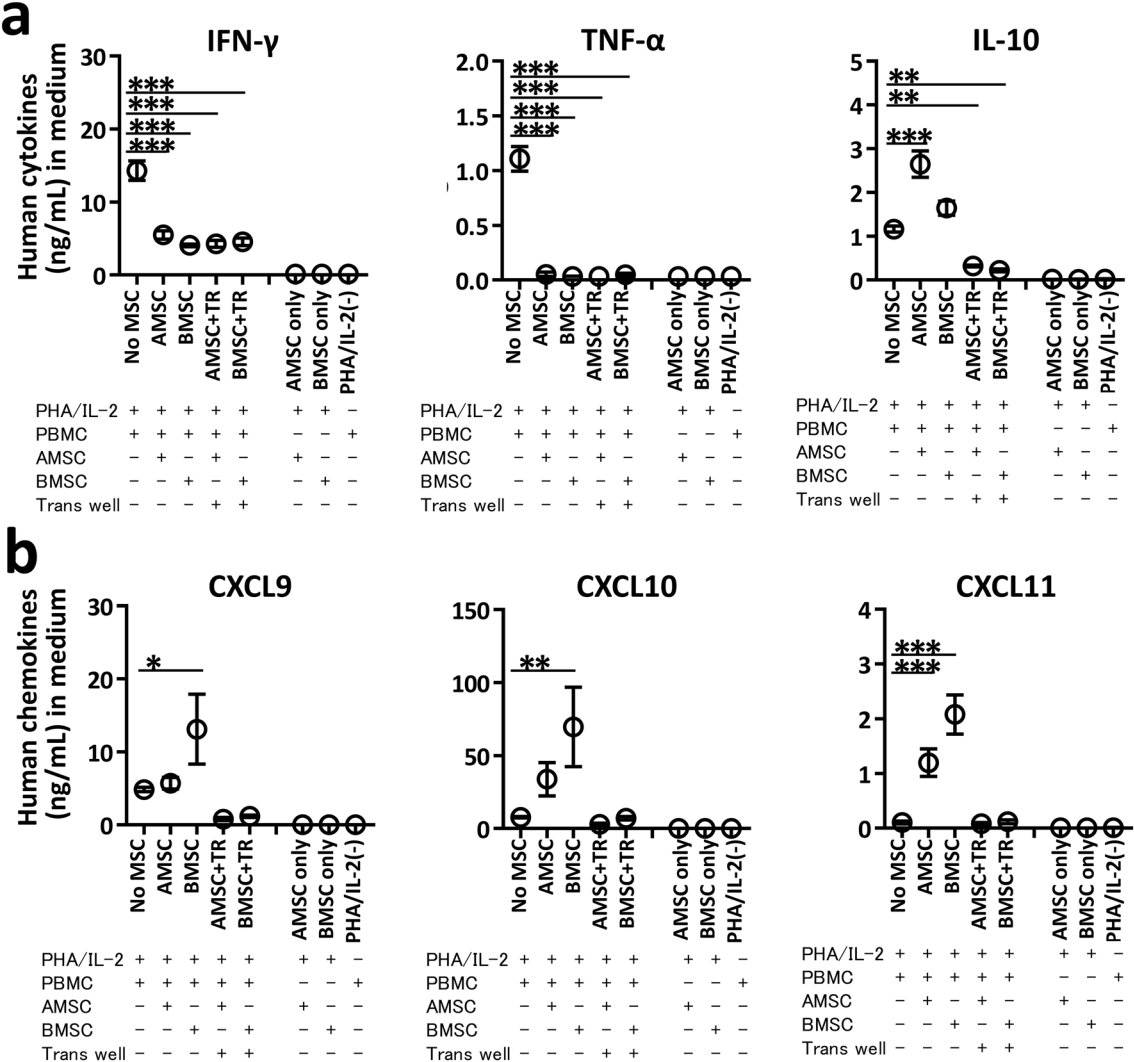
Proinflammatory cytokine and chemokine concentrations in PHA/IL-2 stimulated PBMC in vitro. PBMC were stimulated with 4 μg/mL PHA and 100 U/mL IL-2 for 72 h with or without AMSC or BMSC at a PBMC/MSC ratio of 10:1 (**a**,**b**). For indirect coculture experiments, a trans well (TR) system comprising upper wells with PBMC and lower wells with MSC was used. Combined data from two or three independent experiments are presented as mean ± SEM. (**a**,**b**) Concentrations of human cytokines IFN-γ, TNF-α, and IL-10 as well as human chemokines CXCL9, CXCL10, and CXCL11 in culture medium evaluated by multibead-based immunoassay. * *P* < 0.05, ** *P* < 0.01, *** *P* < 0.001 versus No MSC.

We measured the human proinflammatory cytokine (Fig. 4a) and chemokine (Fig. 4b) concentrations in PBMC–MSC coculture medium. Consistent with the intracellular staining patterns in T cells (Fig. 3c), concentrations of proinflammatory cytokines IFN-γ and TNF-α were significantly reduced in both direct and indirect PBMC–MSC cocultures compared with in No MSC (*P* < 0.001) (Fig. 4a). Concentrations of IL-10, an anti-inflammatory cytokine, were significantly increased in direct PBMC–AMSC cocultures (*P* < 0.001) and decreased in indirect PBMC–AMSC cocultures on the contrary (*P* < 0.01) (Fig. 4a). Surprisingly, concentrations of IL-10 were significantly elevated in only AMSC direct coculture, not BMSC. Concentrations of IL-1β, an inflammatory cytokine, were higher only in direct PBMC–AMSC cocultures, whereas concentrations of IL-6, IL-8, and monocyte chemoattractant protein 1 (MCP-1) were increased regardless of the coculture conditions (Fig. S1). Interestingly, concentrations of proinflammatory chemokines C-X-C motif ligand (CXCL) 9 and CXCL10 were significantly elevated in PBMC–BMSC direct cocultures compared with in No MSC (*P* < 0.05 and *P* < 0.01), whereas these were not significantly changed in PBMC–AMSC direct coculture. Similarly, concentrations of CXCL11 in direct PBMC–BMSC coculture were 1.8 times higher than in direct PBMC-AMSC coculture (Fig. 4b). Additionally, concentrations of C–C motif chemokine ligand (CCL) 20 in direct PBMC–BMSC coculture were 1.9 times higher than in direct PBMC–AMSC coculture, whereas concentrations of CXCL5 was increased regardless of the coculture conditions (Fig. S2).

### PD-L1 and PD-L2 upregulation in AMSC stimulated with IFN-γ/TNF-α is associated with inhibition of T cell proliferation

**Figure 5:**
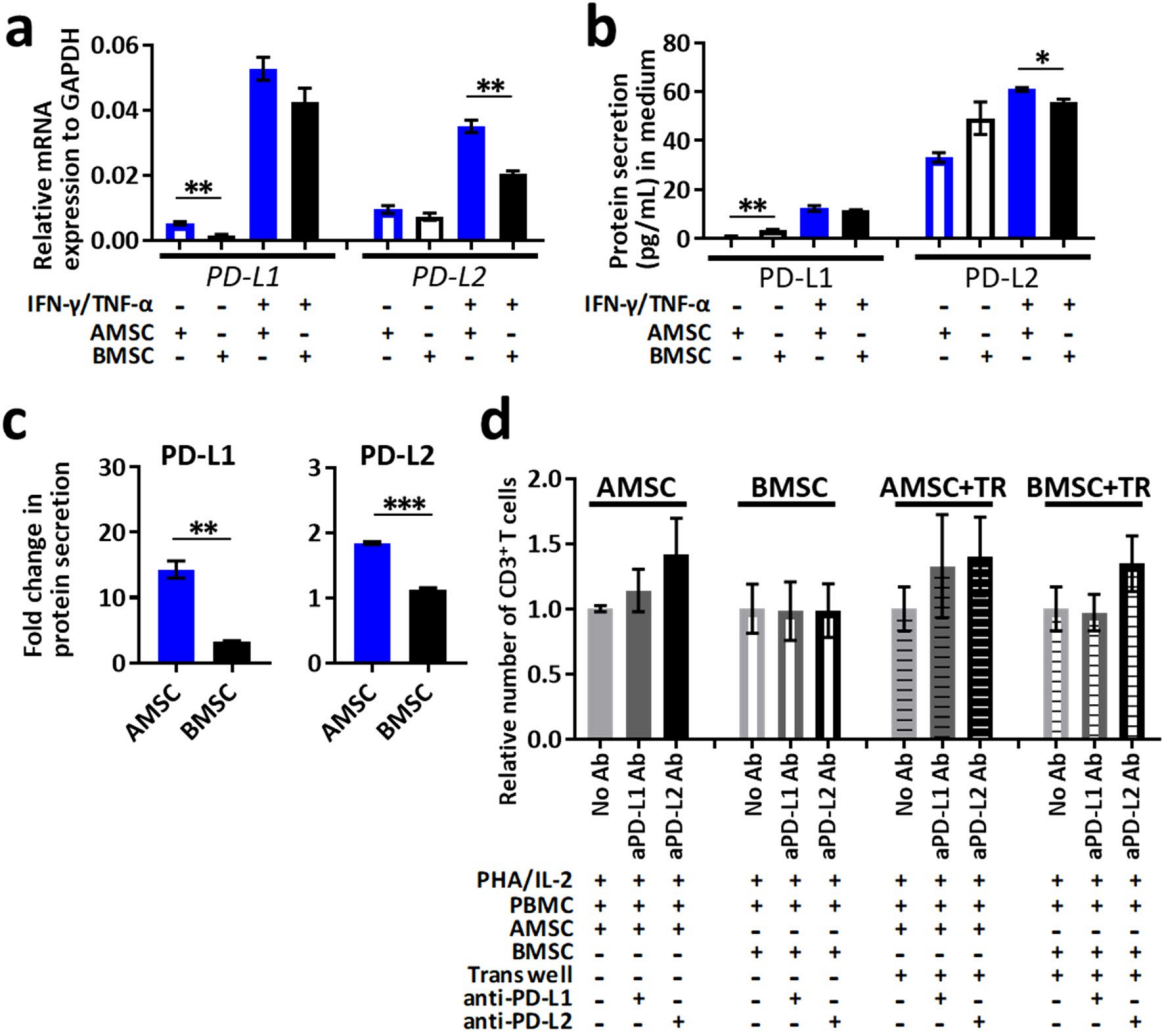
PD-L1 and PD-L2 upregulation in AMSC stimulated with IFN-γ/TNF-α associated with inhibition of T cell proliferation in PHA/IL-2 stimulated PBMC in vitro. (**a**‒**c**) AMSC or BMSC were stimulated with IFN-γ/TNF-α for 24 h. (**a**) Gene expressions of *PD-L1* and *PD-L2* in AMSC or BMSC by real-time PCR. (**b**) Protein concentrations of PD-L1 and PD-L2 in culture medium by ELISA. (**c**) Fold changes in PD-L1 and PD-L2 secretion calculated as the ratio of values in cultures with and without stimulation. (**d**) PBMC were stimulated with 4 μg/mL PHA and 100 U/mL IL-2 for 72 h with or without AMSC or BMSC in the presence of anti-human PD-L1 or PD-L2 antibody (Ab) at a PBMC/MSC ratio of 10:1. For indirect coculture experiments, a TR system comprising upper wells with PBMC and lower wells with MSC was used. Relative number of CD3^+^ T cells shown as 1 for No Ab in each coculture condition. Data from one of two independent experiments (**a**‒**c**) and combined data from more than three independent experiments (**d**) are presented as mean ± SEM. * *P* < 0.05, ** *P* < 0.01, *** *P* < 0.001.

To confirm the immunosuppressive factors secreted by MSC, we treated AMSC or BMSC with IFN-γ and TNF-α, also known as MSC licensing factors^25^ which were shown to be elevated both in vivo and in vitro. Further, we measured PD-L1 and PD-L2, well-known immune modulators secreted by BMSC^26,27^. *PD-L1* and *PD-L2* mRNA expressions were significantly higher in AMSC than in BMSC independent of IFN-γ/TNF-α treatment (*P* < 0.01) (Fig. 5a). Although absolute values of PD-L1 and PD-L2 protein secretions were similar with only slight differences between AMSC and BMSC (Fig. 5b), AMSC had significant difference in the abilities of PD-L1 and PD-L2 secretions during inflammation and non-inflammation compared with BMSC (*P* < 0.01 and *P* < 0.001) (Fig. 5c).

Next, we showed that these ligands could be neutralized by the treatment of MSC with anti-human PD-L1 or PD-L2 antibodies based on ELISA using culture medium (Fig. S3). To evaluate the functional roles of PD-L1 and PD-L2 secreted by MSC against activated T cells, we performed the blocking assays using anti-human PD-L1 and PD-L2 antibodies. Although the results did not show significant differences, it was indicated that the ability to suppress CD3^+^ T cell proliferation by AMSC tended to be weakened in presence of anti-human PD-L1 or PD-L2 antibody both in direct and indirect coculture (Fig. 5d). In other words, T cell activation and proliferation were recovered by the addition of these antibodies. Our data suggested that PD-L2 might be more effective in inhibiting T cell proliferation than PD-L1.

### AMSC can suppress GVHD progression in the early phase of xeno-GVHD mice

**Figure 6:**
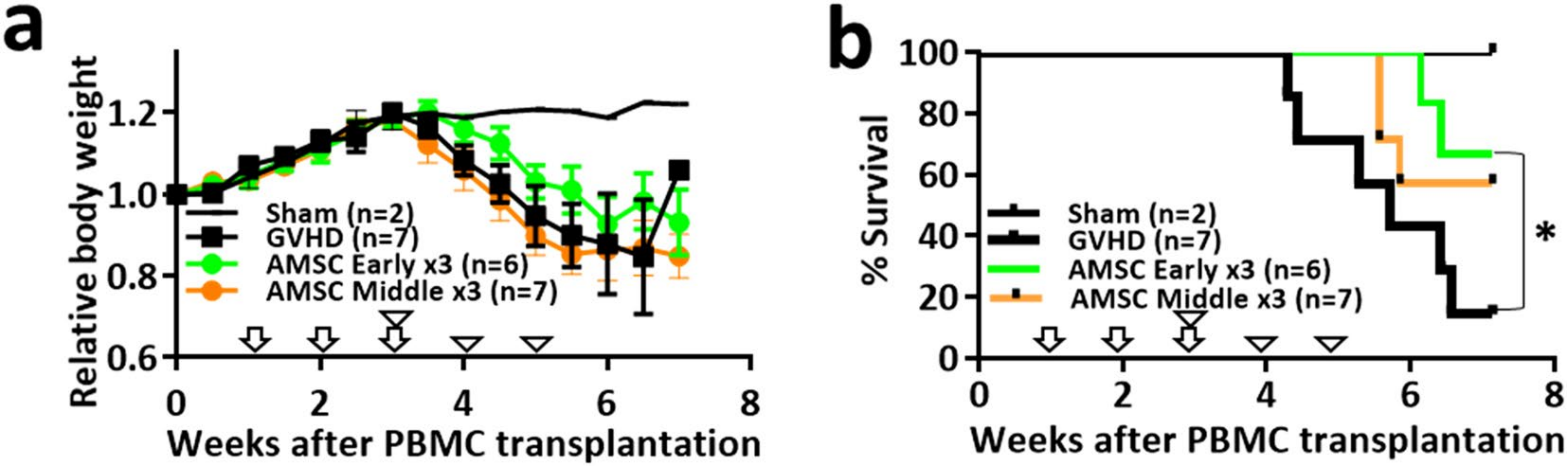
Early-phase administration of AMSC attenuate GVHD progression in xeno-GVHD mice. GVHD was induced by transplantation of 3 × 10^6^ cells/mouse PBMC administered intravenously on week 0. Mice were administered 8 × 10^6^ cells/kg AMSC intravenously three times in the early phase (weeks 1‒3, ↓, n = 6) or middle phase (weeks 3‒5, ▽, n = 7). Sham (n = 2) and GVHD control (n = 7) mice were administered vehicle. Data from one experiment are presented as mean ± SEM. (**a**,**b**) Relative body weight and survival rate. *P < 0.05 AMSC early versus GVHD.

To determine the optimal timing of AMSC delivery to suppress acute GVHD progression, we administered AMSC into xeno-GVHD mice three times in the early phase (weeks 1‒3) or the middle phase (weeks 3‒5). Relative body weight started to decline at around week 3 in all GVHD induction groups (Fig. 6a), and there were no significant differences throughout the experiment. Survival started to decline at around week 4 in GVHD controls, and early AMSC infusion significantly improved mortality compared with that of GVHD controls (*P* < 0.05) (Fig. 6b).

## Discussion

GVHD progresses in three sequential phases: (1) antigen-presenting cells (APC) activation; (2) activation, proliferation, differentiation, and migration of donor T cells; (3) target tissue destruction^1^. Our data suggest that the key roles of AMSC include reduction in PD-1^+^CD8^+^ T cell population (donor T cell activation) and inhibition of colonic inflammation (target tissue destruction) and indicate that AMSC prolong survival of xeno-GVHD mice by delaying disease progression.

We found that AMSC improved colonic inflammation in xeno-GVHD mice, without any changes in liver, spleen, or skin. Onishi et al.^10^ reported that intravenous AMSC administration ameliorated the disease activity index score in rats with dextran sulfate sodium-induced colitis. Low *Tnf-α* mRNA expressions in the rectal tissues of these AMSC-treated rats with colitis coincide with our data showing low TNF-α protein concentrations in blood of AMSC-treated GVHD mice in vivo and mitogen-stimulated PBMC in vitro. Cooke et al.^28^ revealed that TNF-α mediated the development of intestinal inflammation in the early phase of allo-HSCT in mice. We predicted that AMSC attenuated TNF-α producing T cells in the early phase of GVHD in mice, thereby mitigating colonic inflammation. Additionally, other groups have recently demonstrated that the local administration of adipose tissue-derived MSC was potentially therapeutic in perianal fistulizing Crohn’s disease^29^. Therefore, human MSC might have common abilities to suppress gut inflammation. Kamel et al.^30^ demonstrated that IFN-γ secreted by donor cells were higher in the plasma of patients with acute GVHD, consistent with our observations in xeno-GVHD control mice and mitogen-stimulated PBMC controls in the absence of MSC. However, AMSC treatment did not decrease IFN-γ concentrations in vivo, contrary to that observed for TNF-α concentrations.

PD-1 is upregulated on T cells during immune activation^31^. Ahn et al.^32^ reported that CD8^+^ T cells expressed PD-1 early after virus infection, whereas Simonetta et al.^33^ demonstrated that population of CD8^+^ T cells expressing PD-1 increased early in patients undergoing allo-HSCT. We confirmed that percentage of PD-1^+^CD8^+^ T cell population, which was increased over time in xeno-GVHD control mice (similar to that reported in a clinical study^33^), was significantly ameliorated by AMSC infusion.

Weiss et al.^34^ indicated that MSC secrete soluble factors, including prostaglandin E2, indoleamine 2,3-dioxygenase, transforming growth factor beta 1, galectin-1, IL-6, nitric oxide, and PD-L1, to downregulate effector T cells and monocytes. We showed that AMSC and BMSC inhibited CD3^+^ T cell proliferation and PD-1^+^ T cell activation as same levels in vitro regardless of the culture conditions, indicating that contact-independent mechanisms were important.

Conversely, Treg population and concentrations of some cytokines and chemokines were changed in direct cocultures versus in indirect cocultures comprising MSC and PBMC. Typical MSC are well-known to induce Treg by several mechanisms including soluble mediator secretion, cell–cell interactions, and APC modulation^35^. Consistent with our results that Treg populations were more increased in direct PBMC–MSC cocultures, English et al.^36^ reported that cell–cell contact was more important than soluble factors based on an in vitro Treg induction assay using a transwell system. CXCL9, CXCL10, and CXCL11 are ligands of CXCR3, a T cell-specific chemokine receptor associated with chronic inflammatory diseases including hepatitis C virus infection^37,38^. Their concentrations increased in direct PBMC–BMSC coculture, but were markedly suppressed in direct PBMC–AMSC coculture. Because these chemokines induce the migration and activation of leukocytes^39,40^, AMSC might have the ability to decrease proinflammatory chemokines and lead to calm inflammation by contacting stimulated PBMC. On the other hand, concentrations of some proinflammatory cytokines and chemokines were elevated in PBMC–MSC coculture medium. Previously, MSC derived from bone marrow, umbilical cord, adipose tissue, and Wharton’s jelly were reported to increase IL-6 and IL-8 concentrations in coculture with PBMC^25^*.* These changes were similar in that they were partially due to allogeneic response of PBMC versus MSC and should be carefully monitored to avoid the promoting GVHD during clinical setting.

Further, concentration of anti-inflammatory cytokine IL-10 only increased in direct PBMC–AMSC coculture, but not in PBMC–BMSC coculture and indirect coculture. Because Treg populations were not different, AMSC were presumed to be involved in high functionality of Treg. Notably, FOXP3 is expressed not only in Treg but also in naïve CD4^+^ T cells stimulated by mitogens^41^. Additionally, IL-10 concentrations were elevated in mitogen-stimulated PBMC directly cocultured with AMSC in vitro but not in xeno-GVHD mice in this study. Therefore, the differences between xenogeneic and allogenic response in the context of cell–cell contact warrant careful consideration.

Expressions of PD-L1 and PD-L2 were more upregulated in AMSC than in BMSC cultured with IFN-γ and TNF-α, whereas the specific PD-L2 blockade tended to be weakened the immune functions of AMSC in vitro. Davies et al.^26^ reported that PD-L1 and PD-L2 were secreted by BMSC to dampen the AKT pathway, allowing BMSC-mediated suppression of T cell activation. The authors also described that PD-L2 had a 2‒6 fold higher affinity for PD-1 receptor than PD-L1. Further, Survival benefit in AMSC-treated xeno-GVHD mice were maintained although the number of AMSC administrations was reduced from four to three in the early phase but not in the middle phase, indicating that early-phase AMSC infusion was more effective in attenuating acute GVHD progression. Based on our results demonstrating reduction in TNF-α concentrations and percentage of PD-1^+^CD8^+^ T cell population in vivo, together with decrease in T cell proliferation and proinflammatory chemokine concentrations as well as increase in Treg population and IL-10 concentration in vitro, AMSC might have immunomodulative effect related to the inhibition of activation, proliferation, differentiation, and migration of donor T cells, eventually delaying acute GVHD progression.

In this study, highly similar immunosuppressive effects with no significant differences were observed between AMSC and BMSC, except for colonic inflammation and CD8^+^ T cell proliferation in xeno-GVHD mice. In common, Grégoire et al.^20^ reported that administration of MSC from different origin (bone marrow and umbilical cord) slightly prolonged overall survival in xeno-GVHD mice. These results indicated that therapeutic effects did not remarkably differ even though the origin of MSC was different. However, compared to BMSC, AMSC have merits as stem cell source for clinical use include non-invasive procurement, vast abundance of source material and small effect of donor age^42^.

We hypothesized that not only AMSC directly inhibit T cell activation and proliferation, but it also contributes to the suppression of GVHD indirectly by modulating inflammatory-related cytokines and chemokines via the PD-1 pathway. However, immune response observed in xeno-GVHD mice is far from alloreactive GVHD observed in patients, and data to determine the appropriate AMSC dose are lacking. Therefore, future studies are warranted to translate our findings into clinical application.

## Conclusion

To the best of our knowledge, we have provided the first evidence that AMSC exert marked immunosuppression in xeno-GVHD mice in vivo and in mitogen-stimulated PBMC in vitro. We estimated that the therapeutic potential of AMSC was equal to that of BMSC, but AMSC was greater than BMSC in suppressing T cell activation and proliferation. These immunosuppressive effects of AMSC might also be applicable to other immune-related diseases.

## Materials and methods

### MSC

Human AMSC were generated as previously described^12^. Amniotic membranes procured following cesarean section of pregnant females were treated with collagenase and dispase (Sigma Aldrich, MO, USA)^10,12^. Human BMSC from healthy donors were purchased from AllCells (CA, USA). The culture conditions of AMSC and BMSC (i.e., MSC) were determined based on previous studies^43^. In detail, MSC were cultured in MEMα (Thermo Fisher Scientific, MA, USA) supplemented with 1% Antibiotic–Antimycotic (Thermo Fisher Scientific) and 5% human platelet lysate^44^ or 10% fetal bovine serum (Fujifilm Wako Pure Chemicals, Osaka, Japan), and incubated at 37 °C in a humidified incubator at 5% CO_2_. After reaching confluence, spindle-shaped cells were detached using trypsin and cryopreserved in liquid nitrogen until further experiment. AMSC from four donors and BMSC from two donors were used at passages 2‒5. MSC were characterized based on positivity (> 95%) for CD73, CD90, and CD105 and negativity (< 5%) for CD45 by flow cytometry (Figure S4). AMSC had the ability to differentiate into osteocytes and chondrocytes. All experiments were approved by the Ethics Committee of Kaneka Corporation (reference number: 2019–9). Each subject provided signed informed consent, and all procedures were performed in accordance with the relevant local and national guidelines and regulations.

### Xeno-GVHD mouse model

Severe immunodeficient male NOG mice aged 7‒8-weeks were purchased from In-Vivo Science (Kanagawa, Japan). Mice were divided into groups by stratified randomization according to weight. Human PBMC were purchased from HemaCare (CA, USA), and 3 × 10^6^ PBMC/mouse in 0.2 mL phosphate-buffered saline (PBS) were transplanted intravenously via the tail vein in NOG mice on week 0. Body weight was measured twice a week. To investigate the immunosuppressive effects of MSC in xeno-GVHD mice, 8 × 10^6^ cells/kg AMSC or BMSC were administered intravenously once a week for 3‒4 consecutive weeks. Sham control mice were administered PBS instead of PBMC and the cryopreservation vehicle (5% dimethyl sulfoxide and human serum in culture medium) instead of MSC. GVHD control mice were administered PBMC to induce GVHD and the cryopreservation vehicle instead of MSC. Moribund mice and those that survived at the study termination (week 7 in the long-term experiment and week 4 in the middle-term experiment) were sacrificed by exsanguination from the descending aorta under isoflurane inhalation anesthesia (Pfizer, NY, USA). Plasma were cryopreserved at − 80 °C. Liver, skin, colon, and spleen were fixed with 10% buffered formalin and embedded in paraffin. Moribund mice with advanced disease were recorded as deaths. All animal experiments were approved by the Animal Care and Use Committee of Kaneka Corporation (reference number: 2019-7) and performed in accordance with the relevant local and national guidelines and regulations. The study was carried out in compliance with the ARRIVE guidelines.

### In vitro proliferation assay using mitogen-stimulated PBMC

MSC were seeded at a density of 0.6‒6 × 10^4^ cells/mL in 24-well plates. After 24 h, the cells were incubated with 10 μg/mL mitomycin C (Fujifilm) for 2 h at 37 °C to inhibit cell proliferation. Next, 6 × 10^5^ PBMC/mL were added directly or indirectly using a transwell system (pore size, 0.4 μm; Corning, NY, USA) at a PBMC/MSC ratio of 10:1 or 100:1. The lymphocytes in PBMC were stimulated with 4 μg/mL PHA (Fujifilm) and 100 U/mL IL-2 (PeproTech, NJ, USA). Anti-human antibodies against PD-L1 and PD-L2 (AF156 and AF1224; R&D Systems, MN, USA) were used at 250 or 500 ng/mL to inhibit ligand function. PHA/IL-2-stimulated PBMC and MSC were cocultured for 3 days.

### MSC stimulation by inflammatory cytokines

MSC were seeded at 3 × 10^5^ cells/mL in 6-well plates and stimulated with 100 U/mL IFN-γ (R&D Systems) and 100 ng/mL TNF-α (R&D Systems). After 24 h, the cells and supernatants were collected for gene expression and protein secretion analyses.

### Flow cytometry

A small amount of peripheral blood was collected once a week from mouse tail vein using heparinized capillary tubes (Hematlon, Ikemoto Scientific Technology, Kanagawa, Japan). Blood cells obtained from survived mice were processed to obtain single-cell suspensions using High-Yield Lyse (Thermo Fisher Scientific) for preprocessing. The cells were measured by flow cytometer using Attune NxT (Thermo Fisher Scientific) or Accuri C6 (BD Biosciences, CA, USA). Live cells were gated to exclude dead cells based on propidium iodide (Sigma) or LIVE/DEAD fixable dead cell stains (Thermo Fisher Scientific). Monoclonal antibodies against human CD3, CD4, CD8, CD25, FOXP3, IFN-γ, TNF-α, and PD-1 were used. For intracellular staining, 2 μM monensin (Fujifilm) was added for 4 h at 37 °C and cells permeabilized using the FOXP3/Transcription Factor Staining Buffer Set (Thermo Fisher Scientific) according to the manufacturer’s instructions. Data were analyzed using FlowJo software (FlowJo, OR, USA). The antibody information is provided in Table S1.

### Gene expression analysis

Total RNA of MSC was extracted using RNeasy Mini Kit (Qiagen, CA, USA), and reverse transcription was performed using the PrimeScript RT Reagent Kit (Takara Bio, Shiga, Japan). Quantitative real-time polymerase chain reaction was performed using QuantStudio 7 Flex with TaqMan Fast Advanced MasterMix and Taqman Gene Expression Assay (Applied Biosystems, CA, USA). The following primers were used: glyceraldehyde 3-phosphate dehydrogenase *(GAPDH*), Hs99999905-m1; *PD-L1*, Hs00204257_m1; *PD-L2*, Hs00228839_m1 (Applied Biosystems). All data were expressed as the ratio of target gene and *GAPDH* expression.

### Protein analyses

Human PD-L1 and PD-L2 protein concentrations were determined by ELISA (Thermo Fisher Scientific and R&D Systems) according to the manufacturer’s protocols. Optical density was measured at 450 nm by Spectra Max (Molecular Devices, CA, USA). To measure the protein concentrations of multiple human cytokines and chemokines, LEGENDplex multibead-based immunoassays (human inflammation panel and proinflammatory chemokine panel; BioLegend, CA, USA) were used according to the manufacturer’s protocols with minor modifications. Data were analyzed using LEGENDplex software (BioLegend).

### Histopathological analysis

Paraffin sections (4 μm) of mouse tissues were stained with hematoxylin/eosin. GVHD lesions, primarily the extent of inflammatory cell infiltration, were evaluated according to the semiquantitative grading system^19,45^ with minor modifications. Histopathological data from dead mice were basically included. But it was omitted in case of abnormal changes such as gut autolysis. Histopathological score was interpreted by a pathologist: 0 as normal and rare, 1 as focal and slight, 2 as diffuse and mild, 3 as diffuse and moderate, and 4 as diffuse and severe.

### Statistical analysis

Statistical analyses were performed using Prism 7 (GraphPad, CA, USA). Survival data were plotted using the Kaplan–Meier method and analyzed by the log-rank test. The remaining results of in vivo assays were analyzed by the nonparametric, two-tailed, unpaired Mann–Whitney *U* test. In vitro assays were analyzed using two-tailed, unpaired Student’s *t* test, or one-way analysis of variance followed by Dunnett’s test for multiple comparisons. All data were presented as mean ± standard error of the mean. Differences with a *P* < 0.05 were considered statistically significant.

## Supplementary Information

Supplementary Information 1.

## Data Availability

All data generated or analyzed during this study are included in this article, its supplementary information file or are available from the corresponding author on reasonable request.

## References

[1] Ferrara JL, Levine JE, Reddy P, Holler E (2009). Graft-versus-host disease. Lancet.

[2] Murata M (2016). Treatment of acute graft-versus-host disease. Rinsho Ketsueki.

[3] Zeiser R, Blazar BR (2017). Acute graft-versus-host disease—biologic process, prevention, and therapy. N. Engl. J. Med..

[4] Arai Y (2017). Efficacy of antithymocyte globulin for allogeneic hematopoietic cell transplantation: A systematic review and meta-analysis. Leuk. Lymphoma.

[5] Zeiser R (2020). Ruxolitinib for glucocorticoid-refractory acute graft-versus-host disease. N. Engl. J. Med..

[6] Shi Y (2018). Immunoregulatory mechanisms of mesenchymal stem and stromal cells in inflammatory diseases. Nat. Rev. Nephrol..

[7] Le Blanc K (2004). Treatment of severe acute graft-versus-host disease with third party haploidentical mesenchymal stem cells. Lancet.

[8] Najima Y (2017). Mesenchymal stem cells for treatment of graft-versus-host disease. Rinsho Ketsueki.

[9] Magatti M (2008). Human amnion mesenchyme harbors cells with allogeneic T-cell suppression and stimulation capabilities. Stem Cells.

[10] Onishi R (2015). Human amnion-derived mesenchymal stem cell transplantation ameliorates dextran sulfate sodium-induced severe colitis in rats. Cell Transplant..

[11] Miyamoto S (2017). Therapeutic effects of human amnion-derived mesenchymal stem cell transplantation and conditioned medium enema in rats with trinitrobenzene sulfonic acid-induced colitis. Am. J. Transl. Res..

[12] Kobayashi K (2019). On-site fabrication of Bi-layered adhesive mesenchymal stromal cell-dressings for the treatment of heart failure. Biomaterials.

[13] Yamahara K (2014). Comparison of angiogenic, cytoprotective, and immunosuppressive properties of human amnion- and chorion-derived mesenchymal stem cells. PLoS ONE.

[14] Shultz LD, Brehm MA, Garcia-martinez JV, Greiner DL (2012). Humanized mice for immune system investigation: Progress, promise and challenges. Nat. Rev. Immunol..

[15] Poirier N (2012). Preclinical efficacy and immunological safety of FR104, an antagonist anti-CD28 monovalent Fab' antibody. Am. J. Transplant..

[16] Fowler KA (2019). R707, a fully human antibody directed against CC-chemokine receptor 7, attenuates xenogeneic acute graft-versus-host disease. Am. J. Transplant..

[17] Ito R (2009). Highly sensitive model for xenogenic GVHD using severe immunodeficient NOG mice. Transplantation.

[18] Bruck F (2013). Impact of bone marrow-derived mesenchymal stromal cells on experimental xenogeneic graft-versus-host disease. Cytotherapy.

[19] Huang F (2017). Human gingiva-derived mesenchymal stem cells inhibit xeno-graft-versus-host disease CD39-CD73-adenosine and IDO signals. Front. Immunol..

[20] Grégoire C (2019). Comparison of mesenchymal stromal cells from different origins for the treatment of graft-vs.-host-disease in a humanized mouse model. Front. Immunol..

[21] Ito R (2017). A novel xenogeneic graft-versus-host disease model for investigating the pathological role of human CD4 or CD8 T cells using immunodeficient NOG mice. Am. J. Transplant..

[22] Auletta JJ (2015). Human mesenchymal stromal cells attenuate graft-versus-host disease and maintain graft-versus-leukemia activity following experimental allogeneic bone marrow transplantation. Stem Cells.

[23] Ehx G (2018). Xenogeneic graft-versus-host disease in humanized NSG and NSG-HLA-A2/HHD mice. Front. Immunol..

[24] Badia R (2018). CD32 expression is associated to T-cell activation and is not a marker of the HIV-1 reservoir. Nat. Commun..

[25] Kim DS (2018). Enhanced immunosuppressive properties of human mesenchymal stem cells primed by interferon-γ. EBioMedicine.

[26] Davies LC, Heldring N, Kadri N, Le Blanc K (2017). Mesenchymal stromal cell secretion of programmed death-1 ligands regulates T cell mediated immunosuppression. Stem Cells.

[27] Galipeau J, Sensébé L (2018). Mesenchymal stromal cells: clinical challenges and therapeutic opportunities. Cell Stem Cell.

[28] Cooke KR (1998). Tumor necrosis factor- alpha production to lipopolysaccharide stimulation by donor cells predicts the severity of experimental acute graft-versus-host disease. J. Clin. Investig..

[29] Carvello M (2019). Mesenchymal stem cells for perianal Crohn's disease. Cells.

[30] Kamel AM (2019). IL12 and IFNγ secretion by donor mononuclear cells in response to host antigens may predict acute GVHD after HSCT. Immunobiology.

[31] Breton G (2013). Programmed death-1 is a marker for abnormal distribution of naive/memory T cell subsets in HIV-1 infection. J. Immunol..

[32] Ahn E (2018). Role of PD-1 during effector CD8 T cell differentiation. Proc. Natl. Acad. Sci. USA.

[33] Simonetta F (2019). Dynamics of expression of programmed cell death protein-1 (PD-1) on T cells after allogeneic hematopoietic stem cell transplantation. Front. Immunol..

[34] Weiss AR, Dahlke MH (2019). Immunomodulation by mesenchymal stem cells (MSCs): Mechanisms of action of living, apoptotic, and dead MSCs. Front. Immunol..

[35] Ma OK, Chan KH (2016). Immunomodulation by mesenchymal stem cells: Interplay between mesenchymal stem cells and regulatory lymphocytes. World J. Stem Cells.

[36] English K (2009). Cell contact, prostaglandin E (2) and transforming growth factor beta 1 play non-redundant roles in human mesenchymal stem cell induction of CD4+CD25(High) forkhead box P3+ regulatory T cells. Clin. Exp. Immunol..

[37] Zlotnik A, Yoshie O (2012). The chemokine superfamily revisited. Immunity.

[38] Hevezi PA (2011). Gene expression patterns in livers of Hispanic patients infected with hepatitis C virus. Autoimmunity.

[39] Romagnani P, Crescioli C (2012). CXCL10: a candidate biomarker in transplantation. Clin. Chim. Acta.

[40] Ren G (2008). Mesenchymal stem cell-mediated immunosuppression occurs via concerted action of chemokines and nitric oxide. Cell Stem Cell.

[41] Walker MR (2003). Induction of FoxP3 and acquisition of T regulatory activity by stimulated human CD4+CD25- T cells. J. Clin. Investig..

[42] Meesuk L (2016). The immunosuppressive capacity of human mesenchymal stromal cells derived from amnion and bone marrow. Biochem. Biophys. Rep..

[43] Bieback K (2009). Human alternatives to fetal bovine serum for the expansion of mesenchymal stromal cells from bone marrow. Stem Cells.

[44] Mu Y, Wu X, Hao Z (2018). Comparative evaluation of mesenchymal stromal cells from umbilical cord and amniotic membrane in xeno-free conditions. BMC Cell Biol..

[45] Asai O (1998). Suppression of graft-versus-host disease and amplification of graft-versus-tumor effects by activated natural killer cells after allogeneic bone marrow transplantation. J. Clin. Investig..

