# Supplementary material for: Human amnion-derived mesenchymal stem cells attenuate xenogeneic graft-versus-host disease by preventing T cell activation and proliferation
Source: Sci Rep. 2021 Jan 28;11:2406. doi: 10.1038/s41598-021-81916-y (PMC7843654; doi:10.1038/s41598-021-81916-y)
Supplement: Supplementary file 1 — Supplementary Information 1. [file 41598_2021_81916_MOESM1_ESM.pdf]

Human amnion-derived mesenchymal stem cells attenuate xenogeneic graft-versus-host disease by preventing T cell activation and proliferation

Yoshiyuki Tago[1,2], Chiho Kobayashi[2], Mineko Ogura[1], Jutaro Wada[1], Sho Yamaguchi[2], Takashi Yamaguchi[1], Masahiro Hayashi[2], Tomoyuki Nakaishi[2], Hiroshi Kubo[1], Yasuyoshi Ueda[2]

[1]Biotechnology Research Laboratories, Kaneka corporation, Takasago, Japan
[2]Regenerative Medicine and Cell Therapy Laboratories, Kaneka corporation, Kobe, Japan

Correspondence
Yoshiyuki Tago, Ph.D.
Address:1-8, Miyamae-cho, Takasago-cho, Takasago, Hyogo, Japan 676-8688

Supplementary Information

Supplemental Table 1. Antibodies used in flow cytometry.

**Anti-human antibody**	**Clone**	**Manufacturer**
CD3-AlexaFlour700	UCTH1	BioLegend
CD3-PerCP/Cy5.5	OKT3	BioLegend
CD4-AlexaFlour700	SK3	eBioscience
CD4-PerCP/Cy5.5	SK3	BioLegend
CD8-BrilliantViolet510	SK1	BioLegend
CD8-PE/Cy7	SK1	BioLegend
CD25-PE/Cy7	BC96	eBioscience
CD45-APC	REA747	Miltenyi Biotec
CD73-APC	REA804	Miltenyi Biotec
CD90-APC	REA897	Miltenyi Biotec
CD105-APC	REA794	Miltenyi Biotec
CD279(PD-1)-BrilliantViolet785	E12.2H7	BioLegend
IFNγ-AlexaFlour488	4S.B3	BioLegend
TNFα-PE	Mab11	BioLegend
FOXP3-PE	PCH101	eBioscience

**Isotype control antibody**	**Clone**	**Manufacturer**
Mouse IgG1	MOPC-21	BioLegend
Human IgG1	REA293	Miltenyi Biotec
Mouse IgG2	MOPC-173	BioLegend

Supplemental Figures

Supplemental Figure 1

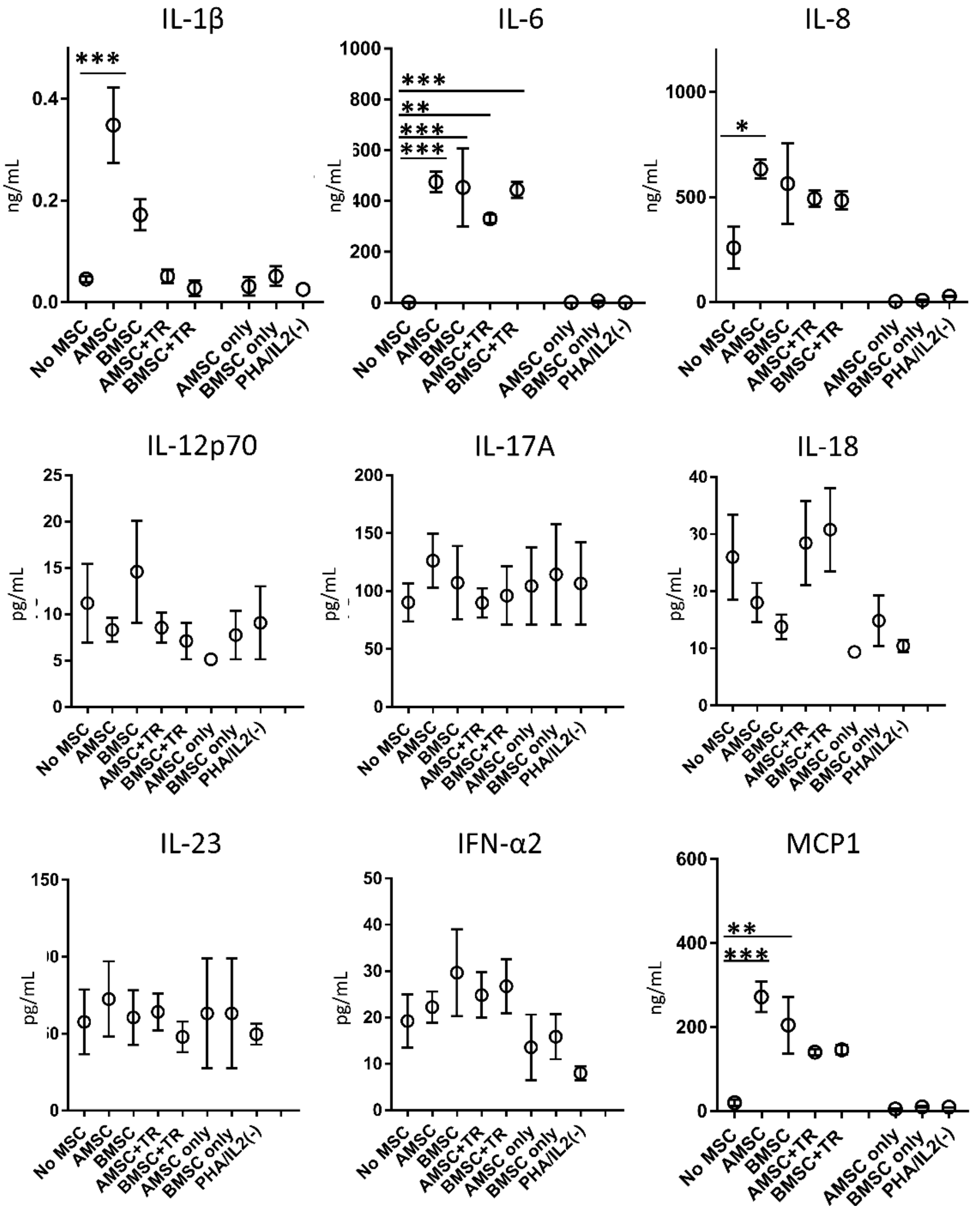

Supplemental Figure 1: Proinflammatory cytokines concentrations not shown in Figure 4a. PBMC were stimulated with 4 µg/mL PHA and 100 U/mL IL-2 for 72 h with or without AMSC or BMSC. For indirect coculture experiments, a TR system comprising upper wells with PBMC and lower wells with MSCs was used. Data from one of two independent experiments are presented as mean ± SEM. Concentration of the human cytokines IL-1β, IL-6, IL-8, IL-12(p70), IL-17A, IL-18, IL-23, IFN-α2, Monocyte Chemoattractant Protein 1 (MCP-1) in culture medium are evaluated by multibead-based immunoassay. The data of IL-33 was not shown because almost values below the detection limit. *P < 0.05, **P < 0.01, ***P < 0.001 versus No MSC.

Supplemental Figure 2

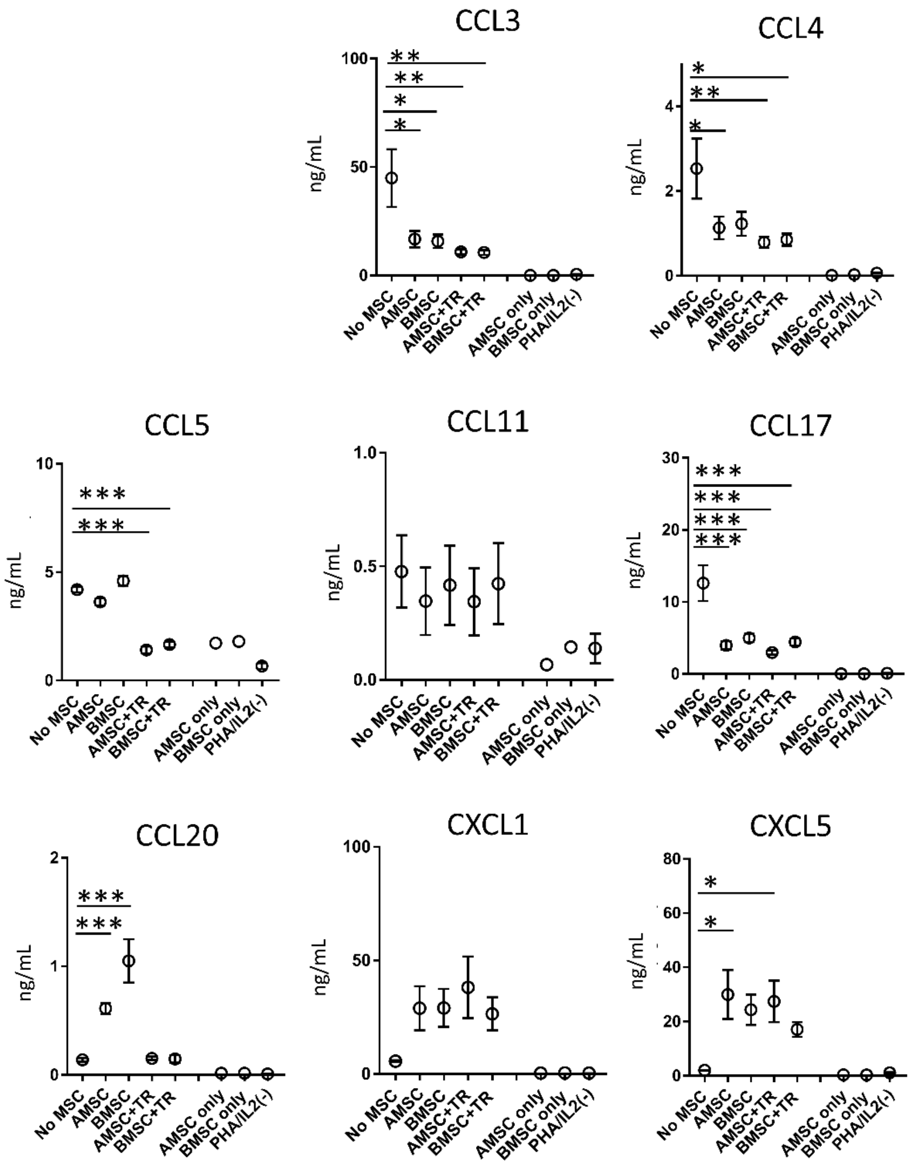

Supplemental Figure 2: Proinflammatory chemokines concentrations not shown in Figure 4b. PBMC were stimulated with 4 μg/mL PHA and 100 U/mL IL-2 for 72 h with or without AMSC or BMSC. For indirect coculture experiments, a TR system comprising upper wells with PBMC and lower wells with MSCs was used. Data from one of two independent experiments are presented as mean ± SEM. Concentration of human chemokines CCL3, CCL4, CCL5, CCL11, CCL17, CCL20, CXCL1, CXCL5 in culture medium are evaluated by multibead-based immunoassay. The data of CCL2 (MCP-1) and CXCL8 (IL-8) were not shown because same items were measured in Figure S1. *P < 0.05, **P < 0.01, ***P < 0.001 versus No MSC.

Supplemental Figure 3

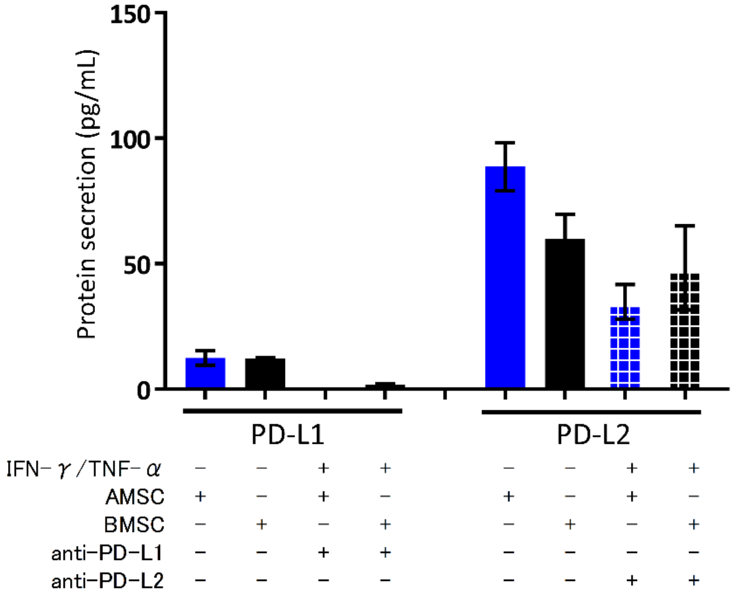

Supplemental Figure 3: Anti-PD-L1 and PD-L2 antibodies are blocked PD-L1 and PD-L2 secretion from AMSC stimulated with IFN-γ/TNF-α. AMSC or BMSC were stimulated with IFN-γ and TNF-α for 24 h including or not including anti-human PD-L1 or PD-L2 antibodies. Data from one experiment are represented mean ± SEM. Protein secretions of PD-L1 and PD-L2 in culture medium by ELISA.

Supplemental Figure 4

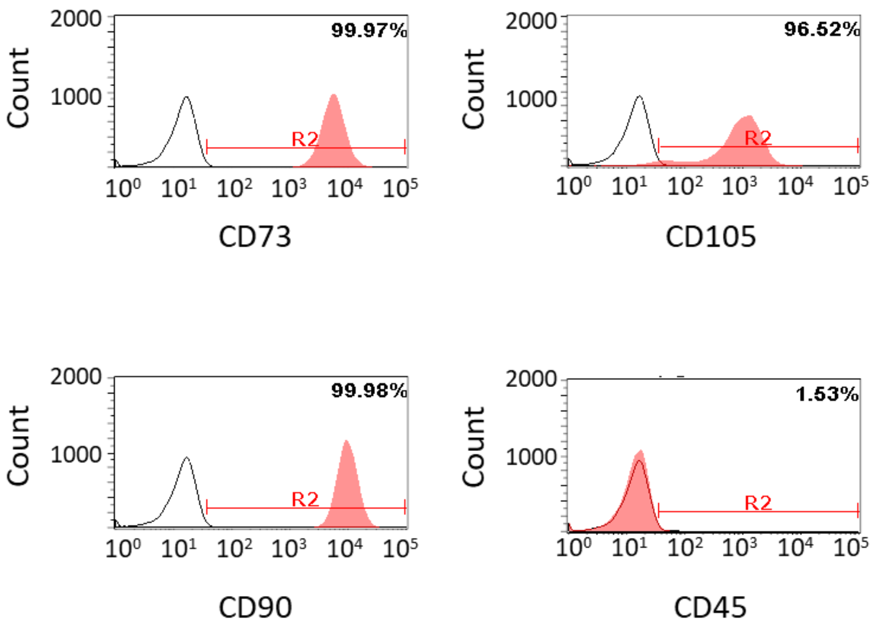

Supplemental Figure 4: Isolated AMSC were characterized for expression of cell surface markers using flow cytometer. AMSC were found to express common MSC cell surface antigens CD73, 90, and 105. AMSC were negative for CD45. Isotype control represented in open histograms.
